# Previous Exposure to an RNA Virus Does Not Protect against Subsequent Infection in *Drosophila melanogaster*

**DOI:** 10.1371/journal.pone.0073833

**Published:** 2013-09-11

**Authors:** Ben Longdon, Chuan Cao, Julien Martinez, Francis M. Jiggins

**Affiliations:** Department of Genetics, University of Cambridge, Cambridge, United Kingdom; Emory University, United States of America

## Abstract

**Background:**

Immune priming has been shown to occur in a wide array of invertebrate taxa, with individuals exposed to a pathogen showing increased protection upon subsequent exposure. However, the mechanisms underlying immune priming are poorly understood. The antiviral RNAi response in *Drosophila melanogaster* is an ideal candidate for providing a specific and acquired response to subsequent infection. We exposed *D. melanogaster* to two challenges of a virus known to produce an antiviral RNAi response, to examine whether any protective effects of prior exposure on survival were observed.

**Results:**

In this experiment we found no evidence that prior exposure to Drosophila C Virus (DCV) protects flies from a subsequent lethal challenge, with almost identical levels of mortality in flies previously exposed to DCV or a control.

**Conclusions:**

Our results confirm the finding that ‘acquired’ immune responses are not ubiquitous across all invertebrate-pathogen interactions. We discuss why we may have observed no effect in this study, with focus on the mechanistic basis of the RNAi pathway.

## Introduction

Over the past decade, a number of studies have demonstrated that invertebrates that have previously encountered a pathogen/parasite appear to be protected upon secondary exposure, which has been termed “immune priming” [1]–[5]. Experimental studies of immune priming have demonstrated that following exposure to either a dead/non-infectious pathogen, or a sub-lethal dose that is subsequently cleared, a host is protected against a later lethal challenge [2], [3], [6]. The effect of immune priming can sometimes even cross generations, with the offspring of infected parents being protected [7]–[10]. While vertebrates produce acquired immune responses to parasites via antibody-mediated immunity, the occurrence of immune priming in invertebrates suggests ‘acquired’ responses are achievable through alternative mechanisms.

Despite the insect innate immune system recognising and killing invaders using receptors and effectors that target molecules conserved across a broad taxonomic range of pathogens, immune priming can sometimes be highly specific [11]. For example, shortly after exposure to three bacterial pathogens, bumblebees initially show a general priming response with little specificity [5]. However, several weeks after the initial infection, the insects only show increased protection on secondary exposure to the bacteria they were previously exposed to, with the priming response even able to distinguish between bacteria from the same genus [5]. Similarly, *Drosophila* primed with *Streptococcus pneumonia* bacteria were protected against a secondary lethal challenge of homologous but not a taxonomically diverse range of other bacteria [12]. In this case it was found that the Toll pathway and phagocytes underlie the increased survival and greater bacterial clearance observed. Specific immune priming has also been reported against different strains of bacterial pathogens in the beetle *Tribolium castaneum* [13] and the crustacean *Daphnia magna* [7], and in the copepod *Macrocyclops albidus* infected with tapeworms [2]. Immune priming has also been shown to occur against double-stranded DNA (dsDNA) viruses. *Penaeus* shrimp with previous exposure to white spot syndrome virus show increased survival on subsequent exposure to virus, with protection persisting for up to two months [14], [15]. Similarly, individuals of the moth *Plodia interpunctella* exposed to a low dose of its natural DNA virus show increased survival on a subsequent challenge to a lethal dose, as do their offspring [10]. Whilst little is known about the mechanisms of specific immune priming in invertebrates, one possibility is the hypervariable immunoglobulin domain-encoding gene *Dscam*. By being alternatively spliced, *Dscam* may be able to produce sufficient receptor diversity to distinguish between different pathogen strains or species [16], [17].

A promising candidate for acquired and specific immunity against viruses is RNAi [18], [19]. The RNAi pathway processes viral double-stranded RNA (dsRNA) into short interfering RNAs (siRNAs), called viRNAs when viral derived [20]. These are then used to guide the cleavage of viral RNA with complementary sequence to the viRNA, resulting in degradation of viral RNA in a sequence-specific manner [19]. This pathway has been shown to protect *Drosophila* from a number of positive and negative sense RNA viruses, as well as a DNA virus [21]. Therefore, RNAi could potentially be a mechanism for highly specific immune priming; if individuals are exposed to a sub-lethal dose of virus that triggers the RNAi pathway, and viRNAs persist, they could confer protection on subsequent exposure to a lethal dose of the same virus.

Rather than the host always clearing a sub-lethal dose of a virus, acute viral infections can develop into persistent infections, where the virus can be present at a low level and cause little damage [22]. RNAi is also important in controlling persistent infections at low levels [23]. In *Drosophila* cells, persistent infections of RNA viruses result in viral RNA being reverse transcribed into chimeric DNA molecules containing retro-transposon and viral sequences [23]. These DNA forms in turn produce transcripts that appear to be processed by the siRNA pathway, and produce viRNAs that inhibit viral replication. Furthermore, inhibiting reverse transcription in adult flies results in greater mortality on viral infection. Along with classical antiviral RNAi, this could provide a mechanism by which low level persistent infections could act to protect flies from subsequent acute viral infection in a manner akin to immune priming (we will define immune priming as being protection following clearance of the initial infection).

To examine whether previous exposure to an insect RNA virus can protect against subsequent infection by the same pathogen, we carried out an experiment using *Drosophila melanogaster* and its naturally occurring pathogen, Drosophila C Virus (DCV). DCV is a positive sense RNA virus in the family *Dicistroviridae* [24] that naturally infects *D. melanogaster* and other *Drosophila* species in the wild [25]–[27]. DCV has been reported to be predominantly horizontally transmitted between individuals in the laboratory, although vertical transmission can also occur [25], [28]. The RNAi pathway has been shown to be an important antiviral defence against DCV, with flies lacking in the major RNAi components showing increased susceptibility [29]–[31], and viRNAs are produced against DCV [32]. In addition to RNAi, the JAK-STAT pathway and a gene called *Pastrel* have been implicated in protecting flies against DCV [21], [33], [34].

Our approach was to expose flies to DCV in two challenges. The first challenge was a low dose that would replicate – and so produce an immune response – but cause minimal mortality. The second was a lethal dose of DCV to see whether the first challenge would protect flies. This is different to the approach in the past priming literature, where the primary infection is dead or rapidly cleared [1], and is designed to resemble the persistent DCV infections that may occur in the wild [35], [36].

## Methods

### Virus Production and Infections

DCV was produced in Schneider Drosophila line 2 (DL2) cells [37] as described in [38]. Cells were cultured at 26.5°C in Schneider’s Drosophila Medium with 10% Fetal Bovine Serum, 100 U/ml penicillin and 100 µg/ml streptomycin (all Invitrogen, UK). Cells were then freeze-thawed twice to lyse cells and centrifuged at 4000 g for 10 minutes at 4**°**C to remove any cellular components or bacteria. Virus was then aliquoted and frozen at −80**°**C. Uninfected cell culture for control solution was produced by growing DL2 cells as for virus production but DCV was replaced with Drosophila Ringer’s solution [39]. To calculate infectivity of the virus, serial dilutions of virus from 10^−1^ to 10^−12^ were carried out in Schneider’s medium, and each dilution was added to 8 wells of a plate of DL2 cells. After 7 days the wells were examined and classed as “infected” when cell death and cytopathic effects were clearly visible. The Tissue Culture Infective Dose 50 (TCID_50_) was calculated by the Reed-Muench end-point method [40].

To infect flies with DCV, a 0.0175 mm diameter anodized steel needle (26002–15, Fine Science Tools, CA, USA) was bent ∼0.25 mm from the end (∼half of the dorsal width of the thorax), dipped in DCV or control solution (uninfected cell culture medium), and the bent part of the needle pricked into the pleural suture on the thorax of flies. A pilot study was used to calculate a suitable sub-lethal dose of DCV (Figure S1). The primary challenge dose (TCID_50_ 2.32×10^6^) used was the maximum non-lethal dose, to try to ensure replication of the virus. The second challenge (TCID_50_ 4.64×10^7^) was a dose that killed ∼85% of flies in a pilot study (Figure S1). The secondary challenge dose was chosen to ensure flies were not simply being overwhelmed by a large viral dose. At doses greater than this other resistance mechanisms – for e.g. *Wolbachia* – are still able to confer protection (Julien Martinez, unpublished data). Virus extract or uninfected cell culture medium was diluted to the desired concentration using Drosophila Ringer’s solution [39].

### Experimental Design

For the main experiment, 3–4 day old female flies were first challenged with the low virus dose (TCID_50_ 2.32×10^6^) or control medium and then challenged again with the high virus dose (TCID_50_ 4.64×10^7^) or control medium, 3 days after the first challenge. Flies therefore fall into four treatments (first-second challenge): control-control, control-virus, virus-virus, virus-control. The 3 day gap between the first and second challenge was designed to allow the first viral challenge to replicate and produce an immune response. Our aim was to produce an immune response that loitered and so conferred protection on subsequent exposure. DCV viral load has been shown to increase from day one post-infection, so we were confident the virus would be replicating and so producing dsRNA [30]. Previous studies have found the RNAi response appears to be active and controlling viral load, with significant differences in viral load between wild-type and RNAi deficient flies, from day one to two post-infection [29], [30].

The experiment was carried out over four days with all the treatments performed on each day and the treatment order randomised within each day. In total we challenged and measured the survival of 2310 flies. Challenged flies were kept in vials of cornmeal medium at 25**°**C and 70% relative humidity. After the second challenge flies were tipped onto fresh cornmeal medium every four days. On average there were 19 flies per vial, with approximately twice the number of vials per treatment when the second challenge was virus (number of flies per treatment: control-control = 377, control-virus = 784, virus-virus = 758, virus-control = 391). Flies were pricked on the opposite side of the thorax for the first and second challenge. As there is a systemic RNAi response in *Drosophila*, inoculating flies in a different location is not expected to alter RNAi-mediated protection [19], [41]. Mortality was recorded daily for 14 days after the second challenge.

The flies used throughout the experiment were from the DrosDel *w* ^1118^ isogenic line kindly provided by Luis Teixeira [37], [42], which was confirmed to be free of DCV and *Wolbachia* before the experiment by RT-PCR as in [38]. Flies were reared in bottles under fixed densities, and flies collected from each bottle were then randomly assigned to treatment groups.

### Measuring Viral RNA Load

To confirm that flies had not become infected with DCV through laboratory contamination during the experiment, and to see whether the first challenge had been cleared or persisted, viral titre was measured by quantitative reverse transcription (qRT-) PCR in flies from the control-control or virus-control treatment respectively on day 14 after the second challenge. Additionally, to confirm whether the first challenge dose replicated, 140 flies were stabbed with the first challenge dose (TCID_50_ 2.32×10^6^), and were then either snap frozen immediately or three days later (7 replicates of 10 flies for each time point).

Flies were snap frozen in liquid nitrogen and immediately homogenised in Trizol reagent (Invitrogen, UK). RNA was then extracted using a chloroform-isopropanol extraction, as described in [43]. The copy-number of viral RNA was measured relative to an endogenous control reference gene (Elongation factor 1α 100E (*EF1α100E*)) using a QuantiTect Virus qRT-PCR kit (Qiagen, UK) and Taqman hydrolysis probes (Sigma-Aldrich, UK) on a BioRad iQ5 thermocycler. Reactions were carried out in duplex with probes using Cy5 (DCV) and FAM (*Ef1α100E*) reporter dyes, following manufactures instructions. Relative viral RNA copy number was then calculated relative to the endogenous control using the ΔC_t_ (critical threshold) method, where the relative viral RNA load is given by 2^−(Virus *Ct*–Control *Ct*)^ assuming 100% amplification efficiency. A dilution series was used to calculate primer efficiencies, which were 104% and 102% for *Ef1α100E* and DCV respectively. The qRT-PCR cycle was: 50**°**C for 20 min, 95**°**C for 5 min then 40 cycles of: 95**°**C for 15 sec, 60**°**C for 45 sec. Two technical replicates were carried out for each of the day 14 control-control and virus-control samples, and a single technical replicate was carried out for the samples looking at the replication of the first challenge dose. For primer and probe sequences see table S1.

### Analysis

The effect of treatment on survival rates was analysed using a Cox’s proportional hazards mixed effect model, which accounted for between-vial variation in survival rates. The hazard for the *i*th individual from vial *j* at time *t* was modelled as:
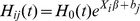



Where *H*_0_ *(t)* is the baseline hazard at time *t*, *X_i_* is a vector of the fixed effects, *β* is the corresponding vector of coefficients, and *b_j_* is a random effect of vial *j* nested within day of the experiment. The fixed effects comprised treatment and day of infection. Flies alive at the end of the experiment were censored. The model was fitted by maximum likelihood using the coxme package [44] in R (R Foundation for Statistical Computing, Vienna, Austria). Data was separated into control or virus secondary treatments so that the assumption of proportional hazards was met. The inclusion of vial as a random effect led to an improvement in the AIC score. The ΔC_t_ values for the qRT-PCR data on viral load were not normally distributed so were analysed using a Wilcoxon Rank Sum test.

## Results

### Survival

**Figure 1:**
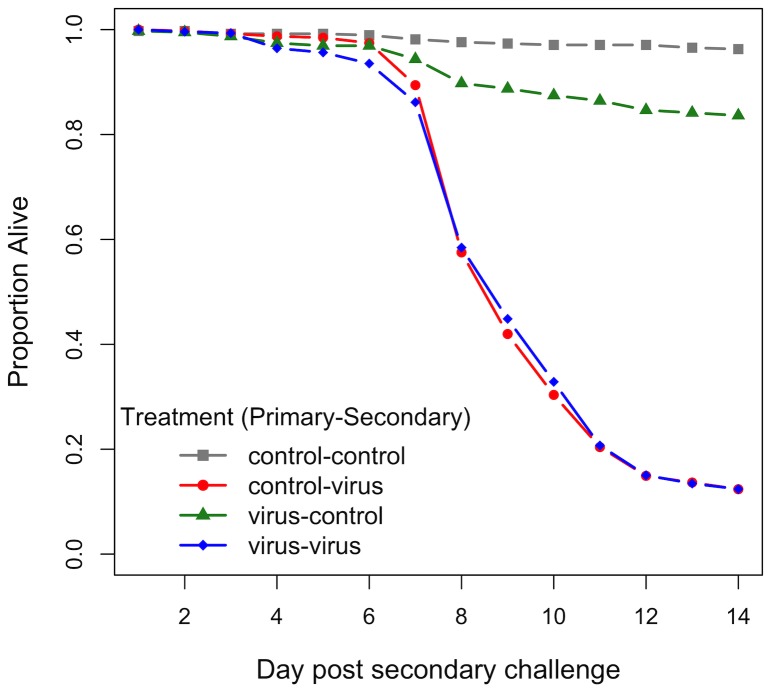
Survival following challenge. Survival of flies inoculated with a low initial dose of DCV or control solution (uninfected cell culture medium) followed by a secondary infection with a high dose of DCV or control solution.

Exposing flies to a low dose of virus had no effect on the rate at which they die when subsequently infected with a larger lethal dose (Figure 1A; z = −0.34, *P* = 0.73). By day 14, both control-virus and virus-virus treatments had 88% mortality. The initial low dose of virus itself caused some mortality, with 16% of the virus-control flies dead on day 14 compared to 4% of control-control flies (Figure 1; z = 3.69 *P*<0.001).

We also examined hazard ratios [45] and their standard errors. The hazard ratios for the control-virus and virus-virus treatments were almost identical with small standard errors (hazard ratio of virus-virus flies relative to control-virus flies = −0.04, SE = 0.11), indicating that our mortality estimates have good precision.

### Viral RNA Load

Our qRT-PCR data confirmed that all 20 samples of day 14 flies (363 flies total) from the control-control treatment contained no detectable viral RNA. This suggests no flies were accidentally contaminated with DCV during the experiment.

The first challenge (low dose) of the virus replicated in almost all samples. Only two of seven samples (each sample consisted of 10 flies) contained detectable amounts of viral RNA immediately after infection, compared to six out of seven samples three days post-infection. There were significantly greater quantities of viral RNA in day three samples (relative viral load from qRT-PCR: day 0 = 0.45 (SE = 0.22), and day3 = 2509.16 (SE = 1529.73), Wilcoxon rank sum test: *W* = 44, *P* = 0.01). By day 14 of the main experiment, 50% of virus-control treated vials contained detectable amounts of virus (10/20, total of 327 flies; mean viral load for infected samples = 110.2, SE = 103.1), suggesting the infection may have been persistent/maintained at low levels in some flies. In vials where DCV RNA was detectable at day 14, there was significantly greater mortality compared to vials where no DCV RNA was detected (24% vs 9% mortality on day 14, Survival analysis: z = 2.64, p = 0.0084, hazard ratio of flies from vials with virus relative to no virus on day 14 = 1.17, SE = 0.45).

## Discussion

We found that in this instance flies previously exposed to DCV were not protected against mortality on subsequent exposure. Given DCV is targeted by the RNAi pathway [29]–[31], we had hypothesised that a sub-lethal challenge could lead to the production of viRNAs that may protect flies against a subsequent lethal infection. It has previously been shown that injection of dsRNA corresponding to DCV can restrict the replication of a subsequent DCV infection due to antiviral RNAi [46]. Given the primary challenge used here appears to have replicated, which should lead to an immune response and the production of dsRNA, why do we not see protection against DCV in this experiment?

There are several possible explanations as to why primary exposure did not protect against mortality during secondary challenge in this experiment. Firstly, DCV is known to encode a viral suppressor of RNAi (VSR) that binds dsRNA and inhibits the production of siRNAs [30]. Therefore, although the first challenge replicated, the VSR may have prevented any prophylactic effect. Giving flies a primary challenge using a DCV strain with a mutated VSR with abolished activity would allow this hypothesis to be tested. A recent study found that the positive sense genomic strand of DCV is the primary target of antiviral RNAi, possibly as a result of the VSR preventing targeting dsRNA replication intermediates [47]. It may also be possible that the approach taken in previous studies of giving a large dose of dead pathogen, or a dose that is rapidly cleared, may have resulted in protection on subsequent exposure.

It may be the case that the immune response against the primary challenge does not persist for long enough to provide prophylaxis. Previous studies in other systems have shown priming can be long-lived [5], [13], [15]. Injection of dsRNA complementary to DCV and Sindbis virus was shown to protect flies against subsequent infection for up to four to five days, but this was shown to be dose dependant for Sindbis virus, with lower doses of dsRNA producing a shorter period of protection [46]. If our primary challenge dose did not produce suitably large quantities of dsRNA, it may be that any response was short lived. The other possibility is the primary dose did not produce sufficient quantities of dsRNA to result in a prophylactic effect on the secondary exposure three days later.

Furthermore, the flies in our experiment may not have produced a systemic antiviral response to the primary challenge, as *D. melanogaster* lacks two mechanisms found in other organisms that are important for generating a systemic RNAi response to viral infection (reviewed in: [19], [41]). Firstly, *D. melanogaster* lacks an RNA-dependent RNA polymerase to amplify dsRNA [48], which can enhance the systemic RNAi response in other taxa [19], [41]. *D. melanogaster* also lacks a transmembrane transporter channel to mediate cell-cell spread of the RNAi response, such as SID-1 that is found in *Caenorhabditis elegans* [49]. Instead, systemic responses to viral infection in *Drosophila* appear to rely on active cellular uptake of dsRNA that is greater than 31 bp long via receptor mediated endocytosis [50], with flies deficient in the uptake of dsRNA showing increased susceptibility to viral infection [46]. However, the proposed mechanism for this is thought to rely on the infected cells releasing viral dsRNAs (through lysis or shedding) that are taken up by uninfected cells (with spread of dsRNAs through the haemolymph rather than active cell-cell transport) [46]. Therefore, a low dose of DCV may not result in sufficient cell lysis or shedding to release suitable quantities of viral dsRNA to trigger a systemic response. As DCV is known to display some tissue tropism [51], the primary exposure in our experiment would have had to stimulate an immune response in these tissues for successful protection on subsequent infection.

Whilst we did not detect any effect of prior exposure in this experiment, it may be the case that under different conditions a protective effect on secondary infection does occur; for example altering the dose and timing of infection may alter the outcome of the experiment. Exposing flies to the virus orally – a more natural route of infection – may also alter the result, perhaps by exposing the gut, which is known to be a preferential site of DCV replication [36], [51]. Similarly, genetic variation in the ability of flies to respond to secondary infection [34], genotype by environment interactions [52]–[54] and coinfection with symbionts [37], [55], [56] or other pathogens [57] may affect the outcome of such interactions.

Immune priming is not a universal phenomenon in invertebrates, with previous studies finding variation in the degree of protection, with some pathogens not eliciting a response [12], [13]. Other studies have found no evidence for priming at all [58]–[60]. Priming can also be variable within a host-parasite system, suggesting it may sometimes be context dependent [10], [61]. Priming can also be costly [62], [63], which may prevent the evolution of priming responses if such costs outweigh the benefits. In this system it may be that such effects have not evolved due to the ecology of the interaction of *D. melanogaster* with DCV in the wild. DCV prevalence in natural populations has been estimated to be low (less than 1%) [26]. Therefore, one could speculate that encounter rates may be too low for an ‘acquired’ response to have evolved [64].

### Conclusions

Immune priming has been demonstrated in a number of invertebrates. We hypothesised that the RNAi pathway could provide an acquired and sequence-specific antiviral immune response. Here, we have examined whether exposure of an insect to an RNA virus results in protection on subsequent exposure. We found no evidence that previous exposure to DCV protected flies from a subsequent lethal challenge in this experiment.

## Supporting Information

Figure S1 **Dilution pilot survival data.** Flies were stabbed with various doses of DCV (TCID_50_ of 4.64×10^9^ to 4.64×10^4)^. Control flies were stabbed with uninfected cell culture medium. Each treatment consists of 3 vials of 20 flies with the exception of the 10^9^ treatment where there were only 2 vials. Following this study, another pilot study was carried out using TCID_50_ of 2.32×10^6^ and 9.28×10^5^ (i.e. 1∶2 and 1∶5 dilution of the previous non-lethal TCID_50_ 4.64×10^5^) to determine the maximum sub-lethal dose. With these doses there were no observable differences in control vs virus treatment at day 10 post-infection.
(TIFF)

Table S1 Primer and Probe sequences used for qRT-PCR.
(DOCX)

Dataset S1 Experimental mortality data, columns are: day of death, day of experiment, whether the primary and secondary challenge was control (C) or virus (V), whether the fly was censored (0 = yes, 1 = no), and the vial identity.
(CSV)
