# Supplementary figures and images for: Previous Exposure to an RNA Virus Does Not Protect against Subsequent Infection in Drosophila melanogaster
Source: PLoS One. 2013 Sep 11;8(9):e73833. doi: 10.1371/journal.pone.0073833 (PMC3770682; doi:10.1371/journal.pone.0073833)

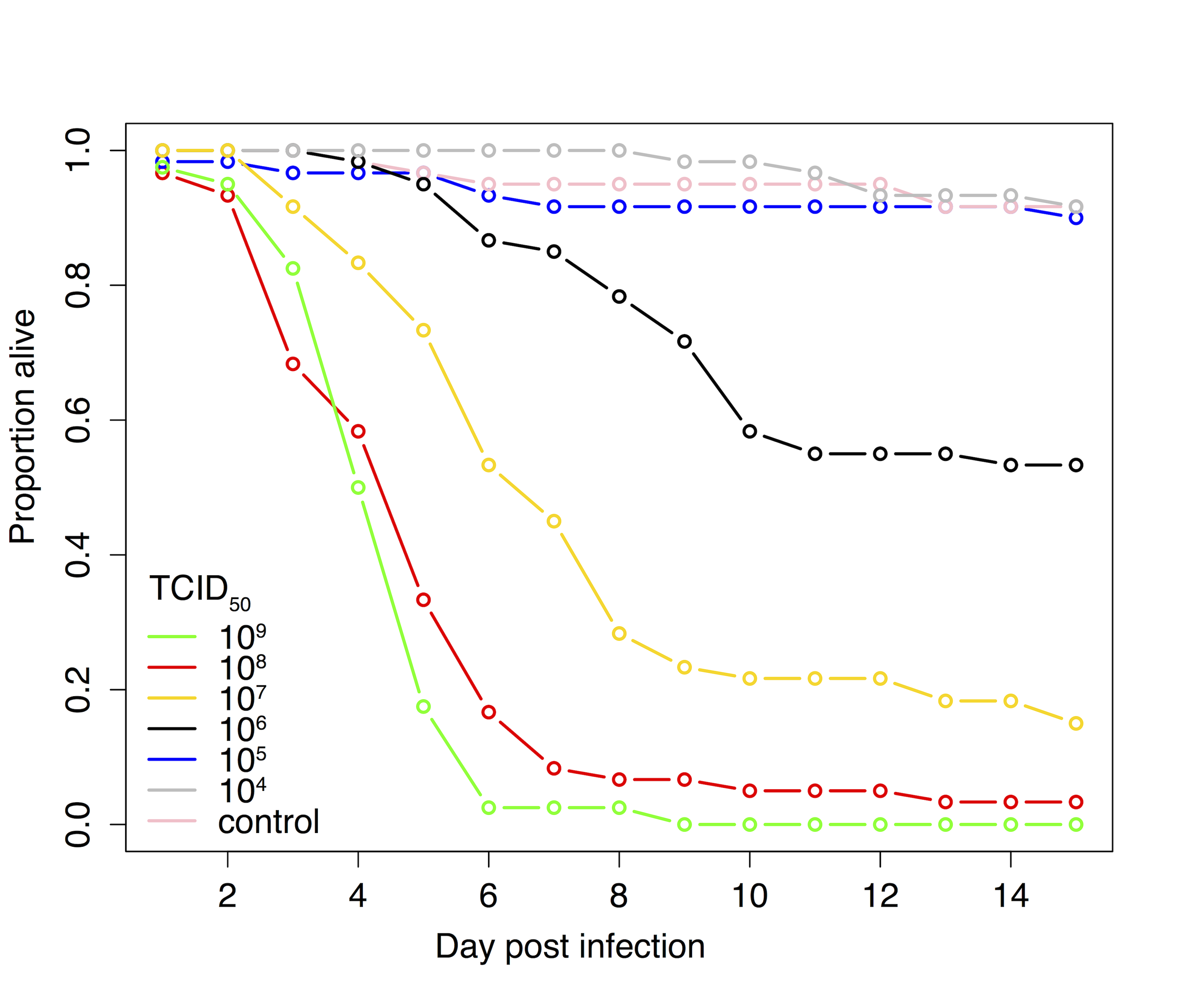

Supplement: Figure S1 — Dilution pilot survival data. Flies were stabbed with various doses of DCV (TCID50 of 4.64×10^9^ to 4.64×10^4^). Control flies were stabbed with uninfected cell culture medium. Each treatment consists of 3 vials of 20 flies with the exception of the 109 treatment where there were only 2 vials. Following this study, another pilot study was carried out using TCID50 of 2.32×10^6^ and 9.28×10^5^ (i.e. 1∶2 and 1∶5 dilution of the previous non-lethal TCID50 4.64×10^5^) to determine the maximum sub-lethal dose. With these doses there were no observable differences in control vs virus treatment at day 10 post-infection. (TIFF) [file pone.0073833.s001.tiff]
