# Supplementary material for: Previous Exposure to an RNA Virus Does Not Protect against Subsequent Infection in Drosophila melanogaster
Source: PLoS One. 2013 Sep 11;8(9):e73833. doi: 10.1371/journal.pone.0073833 (PMC3770682; doi:10.1371/journal.pone.0073833)
Supplement: Table S1 — Primer and Probe sequences used for qRT-PCR. (DOCX) [file pone.0073833.s002.docx]

| Primer/probe | Sequence 5’-3’ |
| --- | --- |
| Ef1alpha100E_F | ACGTCTACAAGATCGGAG |
| Ef1alpha100E_R | CAGACTTTACTTCGGTGAC |
| DCV_F | GACACTGCCTTTGATTAG |
| DCV_R | CCCTCTGGGAACTAAATG |
| Ef1alpha100E_Probe | CATCGGAACCGTACCAGTAGGT |
| DCV_Probe | CACAACCGCTTCCACATATCCTG |
